# Reduced cross-protective potential of Omicron compared to ancestral SARS-CoV-2 spike vaccines against potentially zoonotic coronaviruses

**DOI:** 10.1038/s44298-024-00067-9

**Published:** 2024-11-21

**Authors:** Tyler M. Renner, Matthew Stuible, Brian Cass, Sylvie Perret, Julie Guimond, Simon Lord-Dufour, Michael J. McCluskie, Yves Durocher, Bassel Akache

**Affiliations:** 1 National Research Council Canada, Human Health Therapeutics, Ottawa, ON Canada; 2 National Research Council Canada, Human Health Therapeutics, Montreal, QC Canada

**Keywords:** Protein vaccines, Vaccines, RNA vaccines, Immunology, Adaptive immunity, Humoral immunity, Antibodies, Microbiology, Virology, SARS-CoV-2, SARS virus

## Abstract

The COVID-19 pandemic has emphasised the importance of vaccines and preparedness against viral threats crossing species barriers. In response, a worldwide vaccination campaign targeting SARS-CoV-2 was implemented, which provides some cross-protective immunological memory to other coronavirus species with zoonotic potential. Following a vaccination regimen against SARS-CoV-2 spike in a preclinical mouse model, we were able to demonstrate the induction of neutralizing antibodies towards multiple human ACE2 (hACE2)-binding *Sarbecovirus* spikes. Importantly, compared to vaccines based on the SARS-CoV-2 Reference strain, vaccines based on Omicron spike sequences induced drastically less broadly cross-protective neutralizing antibodies against other hACE2-binding sarbecoviruses. This observation remained true whether the vaccination regimens were based on protein subunit or mRNA / LNP vaccines. Overall, while it may be necessary to update vaccine antigens to combat the evolving SARS-CoV-2 virus for enhanced protection from COVID-19, Reference-based vaccines may be a more valuable tool to protect against novel coronavirus zoonoses.

## Introduction

Historically, a multitude of viruses from different families have exhibited zoonotic potential (e.g., Ebola, influenza, HIV, etc.), however within the last couple of decades, a number of coronaviruses (i.e., SARS-CoV-1, MERS, SARS-CoV-2) have emerged as a particularly significant global threat. While the limited human-to-human spread of SARS-CoV-1 and MERS-CoV restricted their associated outbreaks to epidemic status, the enhanced transmissibility of SARS-CoV-2 played an important role in enabling this virus to expose humanity to a global pandemic, before more recently transitioning to an endemic state^1,2^. The magnitude of damage caused by the COVID-19 pandemic cannot be overstated, directly causing an estimated 7 million deaths to date^3^. Widespread vaccination efforts were able to blunt the impact of the pandemic, due in part to the accelerated production timelines and emergency approval of mRNA- and viral vector-based vaccines, saving millions of lives and reducing health care costs^4,5^. More recently, several alternative vaccine approaches have been developed, with protein subunit vaccines gaining prominence with approvals of Vidprevtyn Beta® (Sanofi) and Nuvaxovid® (Novavax) by the FDA and/or EMA. The efficacy of these various vaccine platforms is tied to their ability to induce neutralizing antibodies against the spike protein, with the chosen protein sequence used (i.e., Reference vs. variant strain) impacting their ability to neutralize infection by a specific variant.

It has been demonstrated that the different SARS-CoV-2 variants exhibit unique immunodominant epitopes that directly influence the antibody repertoire developed post-exposure to each variant^6^. This phenomenon is the most evident in the case of Omicron and its sublineages which have undergone a high degree of mutagenesis from the ancestral SARS-CoV-2 strain, with BA.4/BA.5 spike, for example, having accumulated 28 point mutations and 7 amino acid deletions as compared to the original Reference strain^6^. Over half (17 out of 28) of the point mutations identified in the spike protein of BA.4/BA.5 are located within the receptor binding domain (RBD), which is likely the consequence of viral evolution to escape immunological elimination, resulting in the ability of Omicron spikes to evade neutralizing antibodies induced by prior infections or vaccinations^7–10^. For example, within the RBD of spikes within the Omicron lineage, there are a number of unique mutations within the ACE2 binding motif (e.g., K417N, G446S, E484A and Q493R) that avoid neutralization by the vast majority of potent antibodies found in convalescent serum^8^. Other mutations that were first found in Omicron (BA.4/BA.5), specifically L452R and F486V, which are at the edge of the ACE2 binding interface of the RBD, result in this spike escaping antibodies that were previously shown to effectively neutralize earlier strains of SARS-CoV-2, including Reference and Omicron (BA.1) spikes^10^. These findings support the shift towards modernized monovalent vaccination strategies based on the Omicron antigen sequences as recommended by the World Health Organization and regulatory agencies, resulting in improved neutralization of variants from this lineage^11–13^. In addition, Omicron-based vaccines or infection induce poorly cross-reactive neutralizing antibodies to preceding variants of SARS-CoV-2^14–16^. While the use of modernized antigens should improve outcomes against SARS-CoV-2 infection from the latest circulating strains, it remains to be determined if these modernized vaccines will result in enhanced immunity to other members of the *Sarbecovirus* family.

Given the high probability of further zoonosis events from the coronavirus family, our group sought to investigate the *Sarbecovirus* cross-neutralizing potential of antibodies induced by protein subunit or mRNA/LNP vaccines based on SARS-CoV-2 Reference, Beta, Delta, Omicron (BA.1) and Omicron (BA.4/BA.5) spike sequences.

## Results

We focused on *Sarbecovirus* spikes that bind human ACE2 (hACE2), as the ability of coronaviruses to bind this receptor has been linked to the risk of efficient zoonotic transmission to humans^17^. Also, existing surrogate neutralization assays for SARS-CoV-2 spike can be directly applied to other hACE2-binding spike proteins. Therefore, a subset of hACE2-binding spikes, namely BANAL-20-52, Pangolin-GX, Pangolin-GD, SHC014, Bat-WIV1, and Bat-SARSL sarbecoviruses, were selected for analysis. For the purposes of this study, these spikes have been termed ‘potentially zoonotic’, as an actual zoonosis event has yet to be described for these viruses.

**Fig. 1:**
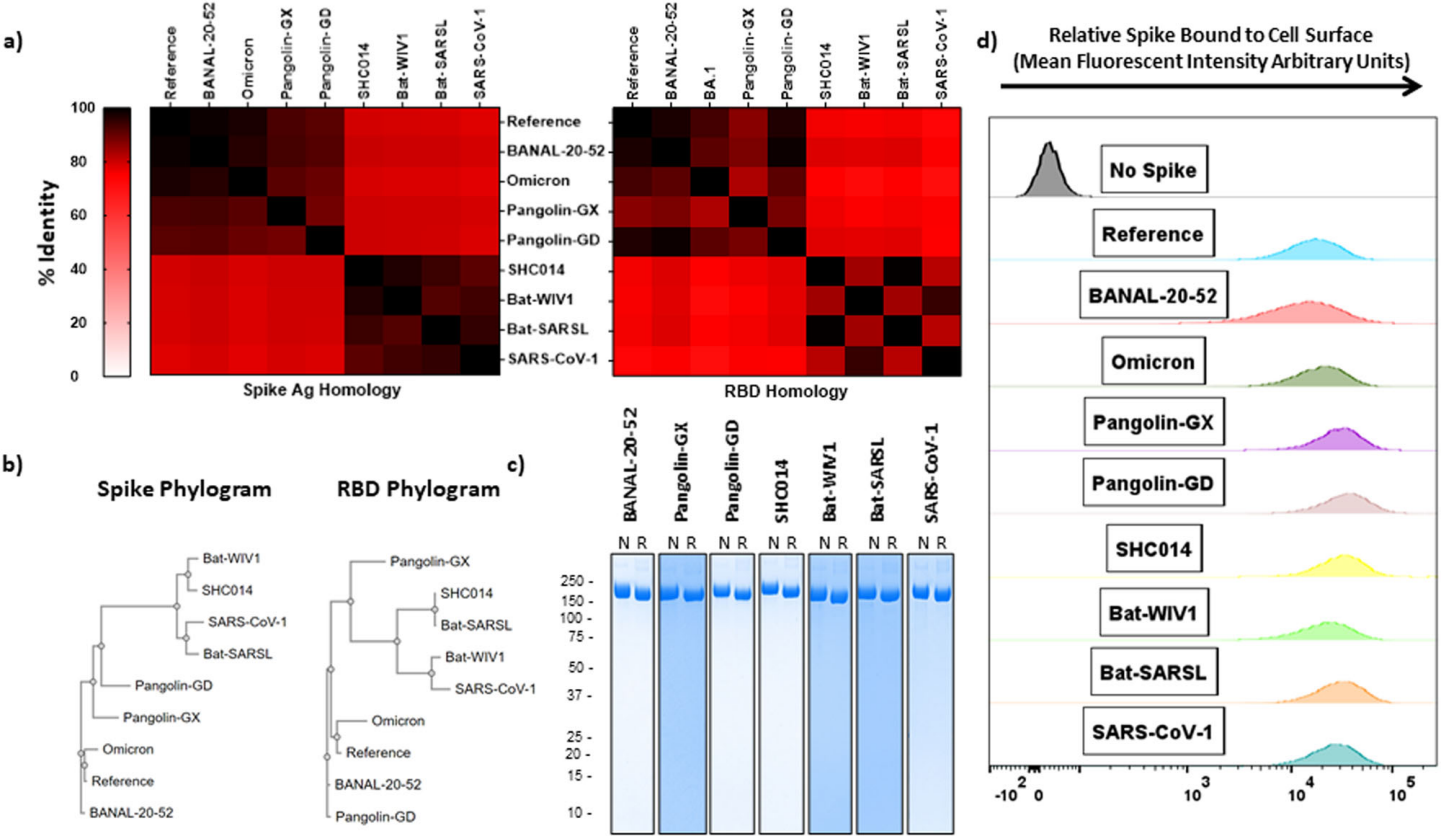
Characterization of hACE2-binding *Sarbecovirus* spike proteins. **a** Heatmap relating the % identity of *Sarbecovirus* spikes (left) or solely based on the receptor binding domain (RBD) sequences (right). **b** Phylograms depicting the points of divergence of the same spike sequences (left) or RBD sequences (right). **c** SDS-PAGE with Coomassie blue staining on purified recombinant *Sarbecovirus* spikes under non-reducing (left) or reducing (right) conditions (numbers along the left side of the gel indicate size in kDa). **d** Interaction of purified spike proteins with HEK293T-hACE2 cells as visualized by flow cytometry. Omicron refers to Omicron (BA.1). RBD Receptor Binding Domain, N Non-reducing, R Reducing.

To illustrate the homology between these spikes and their receptor binding domains (RBD), the amino acid sequences were aligned using Clustal Omega^18^ and the Percent Identity Matrix was plotted in a Heatmap (Fig. 1a). There is a clear divergence of SARS-CoV-1 from SARS-CoV-2, with the *Sarbecovirus* spikes chosen for the current study separating into two groups, being more similar to either of these two SARS viruses. The phylogenetic trees (Fig. 1b) highlight the evolutionary relationship among the tested viral spikes and their receptor binding domains (RBD): based on sequence identity, variants of SARS-CoV-2, such as BA.1, have become more dissimilar from the Reference SARS-CoV-2 spike than some animal-origin viral spikes, such as BANAL-20-52 (97.4% vs 98.7% identity, respectively). These differences are more noticeable in rapidly evolving regions, such as the RBD (93.3% vs 97.3% identity, respectively).

We produced the *Sarbecovirus* spikes using our CHO-expression platform and trimeric antigen design developed previously for the SARS-CoV-2 spike (SmT1)^19^; purity was excellent (>95%) for all constructs with SDS-PAGE/Coomassie staining showing the presence of a single major species at ~170 kDa with low levels of high-molecular-weight bands under non-reducing (left) and reducing (right) conditions (Fig. 1c). We confirmed the ability of these purified spike antigens to bind to HEK293T-hACE2 cells (Fig. 1d), allowing for the assessment of serum cross-neutralization capacity using a flow cytometry-based surrogate neutralization assay established previously^16,20,21^.

Next, using mouse serum generated in previous preclinical studies^16,21^, we evaluated the cross-protective ability of antibodies induced by SARS-CoV-2 spike protein subunit vaccines administered on Days 0 and 21 using equivalent antigen doses (3 µg) of Reference, Beta, Delta, a Trivalent (1 µg each of Reference + Beta + Delta) or Omicron (BA.1) in AddaS03-adjuvanted formulations. Notably, while Omicron (BA.1) has over 30 mutations in the spike protein compared to Reference, the Beta and Delta variants differ by fewer than 10 amino acids. In an earlier study, our group correlated the immunogenicity of this antigen and adjuvant combination to an international World Health Organization (WHO) recognized standard, demonstrating the vaccine formulation induced similar levels of neutralizing antibody responses as those observed in a human vaccination setting with clinically available vaccines standardized to the same metric^21,22^.

**Fig. 2:**
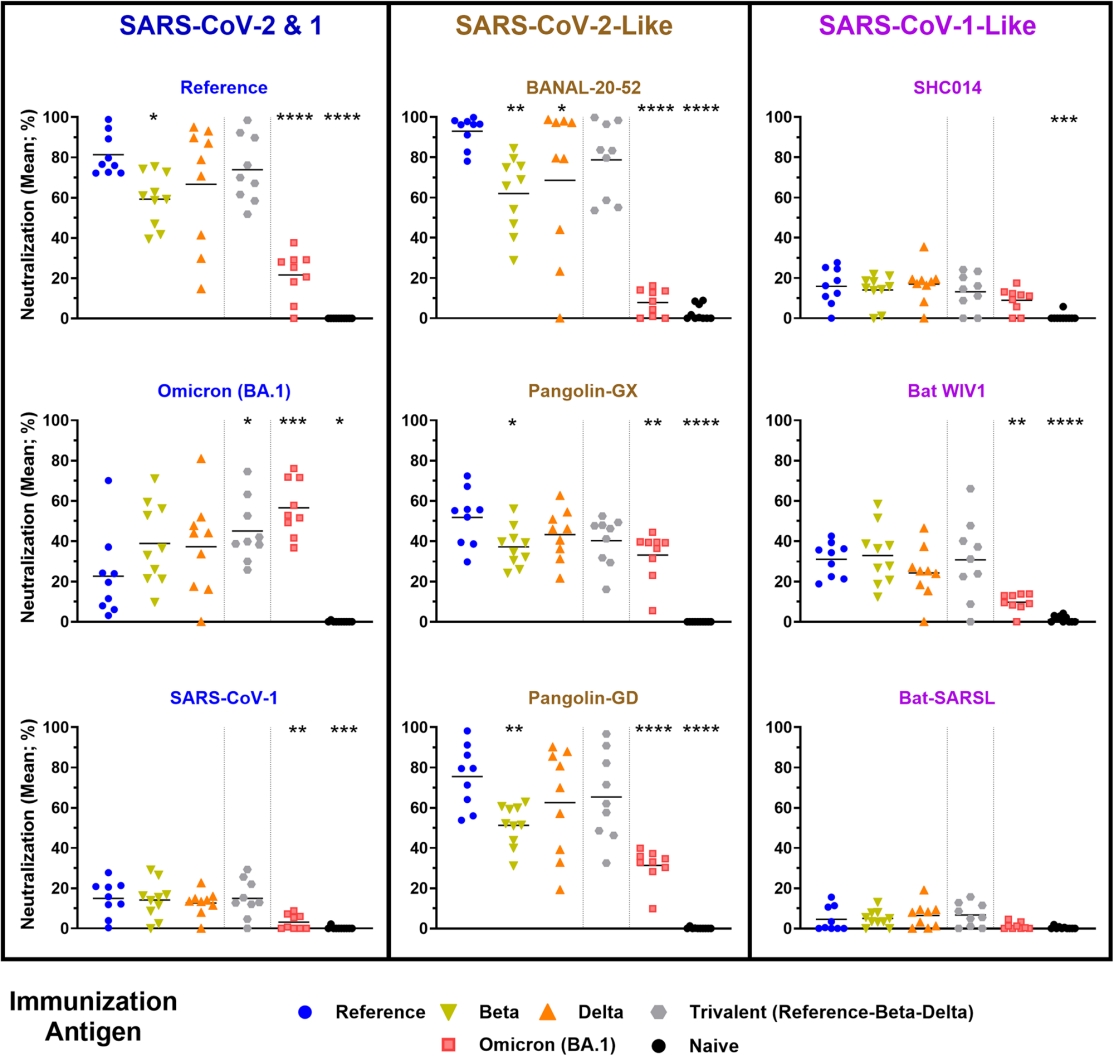
Animal-derived *Sarbecovirus* spikes are better neutralized by serum from mice vaccinated with protein subunit vaccines specific to SARS-CoV-2 Reference than more divergent variants, such as Omicron (BA.1). Neutralizing activity within serum samples from C57BL/6 mice immunized with SARS-CoV-2 Reference, Beta, Delta, Trivalent (Reference-Beta-Delta), or Omicron (BA.1)-based vaccines was assessed using a surrogate cell-based hACE2 binding assay at a dilution of 1:250. Statistical significance of differences among groups vs. the Reference vaccinated mice are shown: *p < 0.05, **p < 0.01, ***p < 0.001 and ****p < 0.0001 by one-way ANOVA followed by Dunnett’s multiple comparison test.

Serum from vaccinated mice was collected on Day 28 (i.e., 7 days post 2^nd^ immunization) for evaluation of neutralization potential against the selected spikes outlined in Fig. 1. This was assessed using a cell-based spike-hACE2 binding assay, which has been previously shown to correlate strongly with the SARS-CoV-2 neutralization activity as determined with other widely-used assays (e.g., PRNT, pseudolentiviral neutralization and in vivo viral challenge^16,20,21,23^). Serum within this assay was diluted 1:250, a concentration which we have previously illustrated enables a suitable dynamic range between biological samples when using this vaccination regimen^21^. Consistent with our previously published data, neutralization activity for a given spike was highest with serum from animals immunized with the matching antigen: Reference and Omicron (BA.1) SARS-CoV-2 were most neutralized by serum from mice vaccinated with Reference (81.3%) and Omicron (BA.1) spike (56.6%), respectively^16,21^ (Fig. 2). The inverse was also true, whereby Reference or Omicron (BA.1) vaccinated mice had the lowest Omicron (BA.1) (22.6%) or Reference (21.6%) neutralizing titer, respectively.

Following this trend, the neutralizability of each spike is linked to their respective relationship to SARS-CoV-2. This is highlighted with BANAL-20-52 and Pangolin-GD, which have a nearly identical neutralization profile to Reference SARS-CoV-2. This correlates strongly with the observed homology within the RBD and not the entire spike protein sequence, as both BANAL-20-52 and Pangolin-GD have ~97% identity to Reference SARS-CoV-2 in this region, while Pangolin-GD has lower (91.2%) homology to the entire Reference spike. Interestingly, serum from the Omicron (BA.1) spike-immunized mice showed much lower neutralization against a number of sarbecoviruses than seen following immunization with Reference and sometimes Beta or Delta spike, which often induced an intermediate neutralizing profile. For example, ~10% neutralization of binding of BANAL-20-52 spike to hACE2 following immunization with the Omicron (BA.1) vs. >90% when the immunization antigen is Reference Spike. Significant differences in the neutralization activity between the sera of Reference and Omicron-immunized mice were also seen against the spike protein based on other SARS-CoV-2-like viruses (Pangolin-GX & Pangolin-GD) as well as SARS-CoV-1 and the SARS-CoV-1-like virus, Bat WIV1. Despite all of these proteins binding the same receptor (hACE2), there was much lower neutralizing activity detected against *Sarbecovirus* spikes that are closely related to SARS-CoV-1. The highest neutralization was observed with Bat-WIV1 spike, with neutralization activity of ~30% seen with serum from mice vaccinated with Reference, Beta or Trivalent SARS-CoV-2 spike, and only ~10% with Omicron (BA.1), at the dilution tested.

**Fig. 3:**
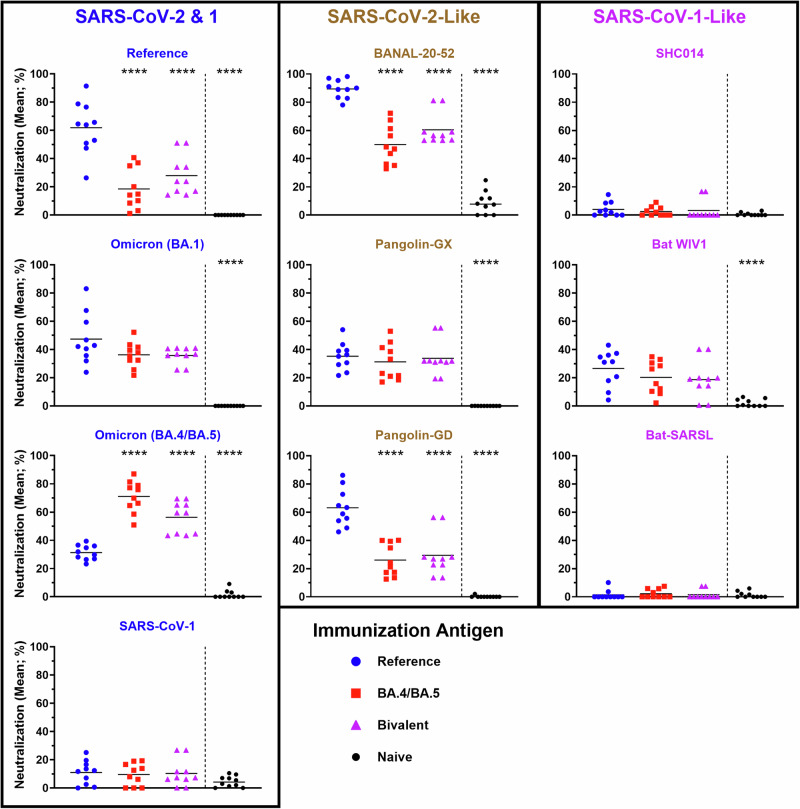
Animal-derived *Sarbecovirus* spikes are better neutralized by serum from mice vaccinated with mRNA/LNP vaccines encoding SARS-CoV-2 Reference than the divergent variant, Omicron (BA.4/BA.5). Neutralizing activity within serum samples from BALB/c mice immunized with SARS-CoV-2 Reference, Omicron (BA.4/BA.5) or Bivalent mRNA/LNP vaccines was assessed using a surrogate cell-based hACE2 binding assay at a dilution of 1:250. Statistical significance of differences among groups vs. the Reference vaccinated mice are shown: ****p < 0.0001 by one-way ANOVA followed by Dunnett’s multiple comparison test.

To confirm the impact of the vaccine platform on these observations, we next evaluated the neutralizing potential of antibodies induced by vaccination with mRNA / lipid nanoparticles (mRNA/LNPs) vaccine formulations encoding different SARS-CoV-2 spikes. We generated DNA templates encoding SARS-CoV-2 Reference and Omicron (BA.4/BA.5) spike proteins, with sequences based on the design used by Pfizer-BioNTech^24^. The mRNA was transcribed in vitro and includes N1-methyl-pseudouridine in place of the canonical uridine, as is typical for the clinically approved mRNA/LNP vaccines^24,25^. After encapsulation into commercially available lipids, BALB/c mice (*n* = 10) were vaccinated with 1 µg of mRNA/LNPs on Day 0 and Day 21. As before, the serum from these animals was assessed 7 days post-boost for *Sarbecovirus* spike neutralizing potential (Fig. 3). As expected, the binding of Reference and Omicron (BA.4/BA.5) spike proteins were most strongly neutralized by serum from mice vaccinated with mRNA / LNPs encoding Reference (61.8%) and Omicron (BA.4/BA.5) spike (71.1%), respectively^16,21^, while the bivalent vaccine induced a more intermediate neutralizing profile. Interestingly, despite containing equivalent doses of each spike mRNA, the Bivalent vaccine induced a more potent neutralizing titer towards the BA.4/BA.5 (56.3%) than the Reference (27.9%) spike. The serum from mice vaccinated with Reference, Omicron (BA.4/BA.5) and Bivalent spike all similarly neutralized (35.8–47.4%) the hACE2-binding of Omicron (BA.1) spike, which has a similar degree of homology to the Reference and Omicron (BA.4/BA.5) spike proteins. As seen with the protein subunit vaccines in Fig. 2, the Reference-based mRNA/LNP vaccines induced significantly higher neutralization profiles of BANAL-20-52 and Pangolin-GD than vaccines designed to target Omicron (BA.4/BA.5). Meanwhile, no significant differences were observed in the neutralization profile of the other spike proteins, including those which had shown less pronounced, but still significant, differences with the protein vaccine formulations (i.e., Pangolin-GX, Bat WIV1 and SARS-CoV-1). Altogether, these data highlight the superior ability of SARS-CoV-2-based vaccine formulations based on the Reference sequence to generate neutralizing antibodies to certain animal-derived sarbecoviruses, whether using protein subunit or mRNA/LNP vaccine platforms.

## Discussion

The COVID-19 pandemic, as well as the relatively recent MERS and SARS outbreaks, have demonstrated the devastating impacts of coronaviral zoonosis and the importance of preparedness to mitigate the impact of similar occurrences in the future. Herein, we sought to better understand whether SARS-CoV-2-based vaccines could potentially protect against future coronaviral zoonotic events. Previous studies have demonstrated that SARS-CoV-1/2 infection and/or vaccination can induce potent neutralizing antibodies with cross-neutralizing potential to certain sarbecoviruses found currently in various animal species^26–32^. Our results confirm that vaccination with formulations based on the ancestral spike of SARS-CoV-2 induces a potent neutralizing response against several hACE2-binding *Sarbecovirus* spikes (e.g., BANAL-20-52, Pangolin-GD, Pangolin-GX, Bat WIV1) that can be superior to those seen with formulations based on variants of concern, in particular Omicron (Figs. 2 and 3). The trends observed herein show a direct relationship between the RBD homology (Fig. 1a) and measured neutralization activity (Figs. 2 and 3). For the current study, a range of hACE2-binding *Sarbecovirus* spike proteins with different degrees of similarity to SARS-CoV-2 were selected for analysis; notably, there are others with zoonotic potential (e.g., BANAL-20-103, BANAL-20-116, BANAL-20-236, BANAL-20-247, etc.) that were not included. Based on our results, inferences can be made about how current vaccines could likely protect against these viruses (based on RBD homology with SARS-CoV-2), but these remain to be confirmed experimentally.

It is important to understand both the positive and negative impacts of updating antigen sequences of SARS-CoV-2 vaccines to match currently circulating variants. The susceptibility of SARS-CoV-2 spikes to neutralization by antibodies generated from exposure to SARS-CoV-2 spike variants with unique immunodominant regions (i.e. Beta, Delta, Omicron, etc.) has been described in detail^6,16,21,33–36^. To our knowledge, no other study has gone further to assess these parameters in the context of potentially zoonotic sarbecoviruses. This can be more complex to measure accurately in human populations due to the heterologous and multifaceted nature of the immune responses generated through previous rounds of infection and/or immunization. Thus, our study focused on a simpler mouse model of homologous prime and boost vaccine regimens. As a recent work illustrated that exposure to an antigen on two separate instances can override pre-existing immune imprinting^35^, the trends observed in these types of models can allow for important insights to be made with regards to how vaccines would perform in more complicated real world scenarios.

In the current study, we used our animal models to compare the cross-protective potential (specifically in terms of induction of *Sarbecovirus*-neutralizing antibodies) of mono- and multi-valent vaccines based on SARS-CoV-2 Reference-strain spike, as well as Beta, Delta and Omicron variants. While all formulations generated similarly weak neutralizing responses to certain spike proteins, namely those more closely related to SARS-CoV-1, they had differing abilities to induce cross-reactive neutralizing antibodies to SARS-CoV-2 variants and related sarbecoviruses. When immunized with protein subunit antigens based on Reference, Beta, Delta or Omicron (BA.1), the least potent cross-neutralizing responses to BANAL-20-52 or Pangolin-GD were observed in mice receiving Omicron (BA.1)-based vaccines (Fig. 2). Similarly poor cross-neutralizing responses were observed to these spike proteins when mice received mRNA/LNPs based on the Omicron (BA.4/BA.5) sequence (Fig. 3), confirming that this effect is dependent upon the antigen sequence and not the platform of vaccination. This may be indicative of “humanization” of the SARS-CoV-2 spike in its more recent iterations (i.e., Omicron) as it has evolved through countless replication cycles in hundreds of millions of infected people. Variants such as Omicron have incorporated critical mutations in their ACE2-binding (and potentially other) motifs to better mediate infection in their new host^6^, simultaneously causing them to diverge away from some of the animal-derived sarbecoviruses tested here, as well as the original SARS-CoV-2 virus that is presumed to have been zoonotically transmitted in 2019. Interestingly, amino acids G446, L452, E484, F486 and Q493 which have mutated in Omicron spike are identical in the spike sequences of Reference SARS-CoV-2 BANAL-20-52, and Pangolin-GD. Uniquely among the spikes tested in this study, BANAL-20-52 shares additional homology at K417, which may partially explain the neutralization profiles observed in this study.

We also found that the tested vaccine candidates induced a lower degree of cross-neutralization against the spike protein of SARS-CoV-1 or its close relatives than seen against the SARS-CoV-2-like sarbecoviruses. This is in contrast to some previous reports that illustrate effective cross-neutralization of SARS-CoV-1-like and SARS-CoV-2-like sarbecoviruses after an initial exposure to SARS-CoV-1^31,37^. This suggests that vaccination with a more closely related *Sarbecovirus* antigen, such as Bat WIV1 or SARS-CoV-1, may be required to effectively neutralize this subgroup of coronavirus spikes. While the overall level of neutralization was lower in the case of the SARS-CoV-1-like sarbecoviruses, for protein antigens, the Reference-based vaccine formulation still induced superior neutralization compared to the Omicron (BA.1)-based antigen against the spike protein from Bat WIV1 and SARS-CoV-1 (Fig. 2). However, for the mRNA/LNP vaccines, no obvious differences were observed between the Reference and Omicron mRNA/LNPs in terms of induction of SARS-CoV-1-related spike neutralization (Fig. 3). Whether this is a feature of vaccination platform (protein vs. mRNA), antigen sequence (BA.1 vs. BA.4/BA.5) and/or mouse strain (C57BL/6 vs. BALB/c) should be confirmed in future studies.

A caveat of this work lies in its direct translatability to the current complex coronavirus-specific immunological landscape in humans. To simplify the design of this study, we did not attempt to mimic the immune imprinting that has occurred in a significant percentage of the human population through a preliminary exposure of the mice to the Reference spike, for example. The first exposure to an antigen will establish the repertoire of the memory immune response, which, due to the speed of memory recall, imposes a bias onto any subsequent immune response to this or similar antigens^38^. Antigenically, infection by one of the several iterations of SARS-CoV-2, including the Reference, Alpha, Beta, Delta and Omicron variants, constituted the first exposure for large proportions of people to the virus antigens^39^, whereas the predominant imprinting immunogen in the vaccinated population would be the Reference SARS-CoV-2 spike^40^. In the case of SARS-CoV-2 vaccination in humans, the effect of imprinting is highlighted by the poor generation of Omicron neutralizing antibodies following a single boost with Omicron-containing vaccines, despite the strong induction of IgG recognizing the Reference spike^41–43^. Therefore, relative to the results seen in a non-imprinted mouse model presented herein, the persistent baseline of Reference spike immunity in the human population may enhance the cross-protective potential of any concurrent SARS-CoV-2 vaccine towards closely related sarbecoviruses, such as BANAL-20-52 and Pangolin-GD. However, as SARS-CoV-2 vaccines are continually updated to keep up with the ongoing viral evolution, one would expect the breadth of protection to decline. Any implications of SARS-CoV-2 imprinting would have a lesser or negligible impact on viral strains with spikes that are sufficiently divergent from SARS-CoV-2 (e.g., Bat-SARSL). Nonetheless, given the ability of a prime and boost vaccination regimen to overcome immune imprinting within mice and humans^35^, the trends observed herein warrant further investigation. Another consideration for these future studies would be to attempt to account for the development of hybrid immunity, wherein vaccine recipients have also been infected at varying timepoints. For this purpose, the use of human serum would be ideal, as it may prove impossible to use a mouse model to properly emulate the hybrid immunity that has been developed in the human population through multiple vaccinations and infections at variable points in time^12,44^.

SARS-CoV-2 Reference-based vaccines have continuously proved to be effective boosters even in the context of emerging variants^45,46^. Nonetheless, it is increasingly evident that updating of SARS-CoV-2 vaccine antigen sequences to resemble dominant, circulating variants is necessary to achieve optimal protection^8,35,47,48^. Importantly, our results indicate that these modernized vaccines may be less capable of inducing protective humoral responses towards other *Sarbecovirus* spikes which have the potential to transmit zoonotically. Furthermore, our data in Fig. 3 illustrates that the use of bivalent vaccine strategies may not induce neutralizing antibodies to both antigens equally, as Omicron (BA.4/BA.5) appears to be immunodominant. This immunodominance over the Reference spike has also been observed previously even when limiting the vaccine to the RBD region^49^.

As highlighted with COVID-19, the swift timing of vaccine deployment is critical to minimize the global impacts at the onset of any pandemic^4,5^. With its cross-neutralizing potential against closely related sarbecoviruses, the ability to quickly deploy a stockpile of Reference SARS-CoV-2 vaccines may prove effective in the event of another outbreak caused by a zoonotic transmission event involving an ACE2-binding coronavirus. The same may be true for other coronaviruses, such as those of the SARS-CoV-1 and MERS-CoV families. Capabilities should be established to quickly develop/manufacture and/or stockpile vaccines based on one (or a few) viruses in their respective families, as they may prove to be effective during an emergency response to a future outbreak. Indeed, our results suggest that with the current immunological status of the overall human population following infection by/vaccination against SARS-CoV-2, viruses from other subgroups may now have a higher likelihood of zoonoses than sarbecoviruses closely related to SARS-CoV-2. In response to a future coronavirus outbreak, this strategy could provide some level of protection during the time delay required for viral identification, de novo synthesis of antigen sequences and regulatory approval of novel vaccines.

## Materials and methods

### Antigen production and homology

CHO codon-optimized sequences encoding spike ectodomains of animal-origin sarbecoviruses (with prefusion-stabilizing 2P mutations) fused to human resistin and purification tags (FLAG-dual-Strep-6His or dual-Strep-6His) at the C-terminus were synthesized at Genscript and cloned into the pTT241® plasmid^50^. These stabilizing prolines are in well-conserved regions for all the selected spikes and were positioned by alignment with the SARS-CoV-2 spike sequence. To mediate trimerization, human resistin was fused to the C-terminus followed by purification tags. Stably transfected pools were generated using CHO^2353^™ cells and protein productions were performed with expression induced with cumate as described^50^. For the different *Sarbecovirus* spikes, protein expression using stably transfected CHO cell pools generated quite variable production yields ranging from 0.1 to 2 g/L. In comparison, the corresponding SARS-CoV-2 Reference-strain construct yields ~0.7 g/L under the same conditions^50^. Clarified CHO culture supernatants harvested at 7 or 10 days post-induction were purified as described by IMAC followed by Strep-affinity chromatography^19^. Purified products were buffer-exchanged using Centripure 100 desalting columns (emp BIOTECH, Berlin, Germany) into DPBS, pH 7.8, or into HEPES (50 mM, pH 7.8) buffer containing 100 mM NaCl. Purified proteins were analyzed by SDS-PAGE using NuPAGE 4–12% Bis-Tris gels (ThermoFisher, Massachusetts, USA) followed by Coomassie Blue staining. The absence of endotoxin contamination was verified using Endosafe cartridge-based Limulus amebocyte lysate tests (Charles River Laboratories, Charleston, SC, USA). Clustal Omega^18^ was used to generate the Percent Identity Matrix and Phylograms to depict the relationship of the spike sequences used in this study. To better relate identity to immunological data, the antigen sequences (which are limited to the spike ectodomains) were used for the homology analyses.

### Preparation of mRNA/LNPs

The plasmid DNA templates used to generate the tested mRNAs were designed based on the publicly available Pfizer-BioNTech mRNA/LNP vaccine sequence^24^ and synthesized by Genscript (Piscataway, NJ, USA) in a pUC57 backbone. DNA templates were linearized with XbaI and purified by phenol–chloroform extraction. RNA was generated using Megascript T7 transcription kit (Thermo Fisher Scientific, Waltham, MA, USA) and capped using CleanCap Reagent AG (TriLink BioTechnologies, San Diego, CA, USA) to generate RNA with stabilized Cap 1 structure. In place of uridine, m1ψ (TriLink BioTechnologies) was included to generate the mRNAs with modified nucleotide chemistry. Following IVT of RNA, plasmid DNA was digested using Turbo DNase (Invitrogen, Waltham, MA, USA) as per manufacturer’s instructions. RNA was purified by lithium chloride precipitation, washed with 70% ethanol and resuspended in RNase-free water. To assess RNA size and integrity, all mRNA samples were resolved on formaldehyde (2.6%, v/v) denaturing agarose (1%, w/v) gel. The concentrations of mRNA samples were measured using Invitrogen’s RiboGreen assay reagent (Thermo Fisher Scientific), as per manufacturer’s recommendations.

IVT mRNA encoding SARS-CoV-2 Reference or BA.4/BA.5 spike was encapsulated within Genvoy-ILM ionizable lipid mixture (Precision NanoSystems, Vancouver, BC, Canada) at an N:P ratio of 6. Formation of LNPs was achieved by microfluidic mixing of RNA (aqueous) and ionizable lipids (organic) using the NanoAssemblr Ignite system (Precision NanoSystems) according to manufacturer’s recommendations with a flow rate ratio of 3 (aqueous:organic) and total flow rate of 12 (ml/min). Formulations were then diluted in Mg^2+^/Ca^2+^-free PBS, before undergoing buffer exchange and concentration using Amicon Ultra Centrifugal Filters (Millipore Sigma, St. Louis, MO, USA). Encapsulation efficiency of RNA was determined by RiboGreen assay (Thermo Fisher Scientific) as per instructions accompanying the Genvoy-ILM ionizable lipid and found to be >90%. In addition, particle size and polydispersity index (PDI) of the LNPs were verified using a Zetasizer NanoZS (Malvern Instruments, Malvern, UK) and found to be 122–136 nm and 0.10–0.12, respectively.

### Immunizations and sample collection

Female C57BL/6 or BALB/c mice (6–8 weeks old) were obtained from Charles River Laboratories (Saint-Constant, QC, Canada) and maintained at the small animal facility of the NRC Canada in accordance with the guidelines of the Canadian Council of Animal Care. Protein subunit vaccinations (Fig. 2) were described in previously published studies^16,21^. Briefly, C57BL/6 mice (*n* = 9–10 per group) were immunized by i.m. injection (50 μl) into the left tibialis anterior muscle on Days 0 and 21 with 3 μg SARS-CoV-2 spike SmT1 antigen (Reference, Beta, Delta, a 1:1:1 ratio of each Reference, Beta and Delta, or Omicron (BA.1)) diluted in PBS (Spectrum, Gardena, CA, USA) and mixed with AddaS03 (Invivogen, San Diego, CA, USA) according to manufacturer’s instructions. Vaccinations of BALB/c mice (*n* = 10 per group) with mRNA/LNPs (Fig. 3) were performed in the same fashion as the protein subunit vaccines except normalized to a 1 μg dose of encapsulated mRNA encoding SARS-CoV-2 spike (Reference, Omicron (BA.4/BA.5) or a 1:1 ratio of each). Mice were bled via the submandibular vein on day 28 with recovered serum used for quantification neutralization activity. Samples were simultaneously collected from 10 naïve animals for the assessment of background immune responses. Each of the samples from the individual mice was tested separately in the various readouts.

### Cell-based spike-hACE2 binding assay

Serum was assessed for its ability to neutralize the binding of labeled spike trimers to hACE2-expressing cells. The following reagent was obtained through BEI Resources, NIAID, NIH: Human Embryonic Kidney Cells (HEK-293T) Expressing Human Angiotensin-Converting Enzyme 2, HEK-293T-hACE2 Cell Line, NR-52511. This assay was performed as previously described, using HEK-293T-hACE2 cells^16^. Purified spike was biotinylated with EZ-Link™ Sulfo-NHS-LC-LC-Biotin (ThermoFisher, Massachusetts, USA) and purified via molecular weight cut off (MWCO) columns according to manufacturer’s instructions. Mouse serum was diluted 1 in 250, mixed with 250 ng of biotinylated spike and 1 × 10^5^ HEK-293T-hACE2 cells. The amount of bound spike was quantified using Streptavidin-R-PE (ThermoFisher, Massachusetts, USA), conjugate by acquiring cells on an LSR Fortessa (Becton Dickinson, New Jersey, USA) and analyzing data on FlowJo (Becton Dickinson). For illustration/analysis purposes, samples with calculated values ≤ 0 were assigned a value of 0. The 1 in 250 serum dilution was chosen here based on previous results from multiple studies. Results generated in this assay with similar serum dilutions show a strong correlation to responses obtained with viral-based SARS-CoV-2 assays, namely viral PRNT (plaque reduction neutralization titer) and 50% neutralization titer of SARS-CoV-2 pseudolentivirus^16,20,21,23^.

### Statistical analysis

Data were analyzed using GraphPad Prism version 10 (GraphPad, Massachusetts, USA). Statistical significance of the difference between groups was calculated by one-way ANOVA followed by Dunnett’s multiple comparisons test. Differences were considered to be not significant with *p* > 0.05. Significance was indicated in the graphs as follows: *p < 0.05, **p < 0.01, ***p < 0.001, and ****p < 0.0001.

## Data Availability

The original contributions presented in the study are included in the article/supplementary materials, further inquiries can be directed to the corresponding author/s.
